# Cellular Distribution and Expression of Cannabinoid Receptors Type 1 and 2 in the Urinary Bladder of Cats (*Felis catus*)

**DOI:** 10.3390/ijms27219910

**Published:** 2026-11-05

**Authors:** Rodrigo Zamith Cunha, Augusta Zannoni, Giulia Biondolillo, Chiara Bernardini, Lia Nasi, Chiara Di Placido, Roberto Chiocchetti

**Affiliations:** 1 Department of Veterinary Medical Sciences, University of Bologna, 40126 Bologna, Italy; augusta.zannoni@unibo.it (A.Z.); giulia.biondolillo2@unibo.it (G.B.); chiara.bernardini5@unibo.it (C.B.); roberto.chiocchetti@unibo.it (R.C.); 2 Department of Veterinary Medicine, University of Teramo, 64100 Teramo, Italy; 3 Independent Researcher, São Paulo 01307-000, Brazil; lia.nasi@studio.unibo.it; 4 RI.MOS Medical Products, 41037 Mirandola, Italy; chiarawolf@live.it

**Keywords:** detrusor muscle, endocannabinoid system, FLUTD, umbrella cells, urothelium

## Abstract

This study aimed to qualitatively and quantitatively characterize the gene and protein expression of cannabinoid receptors type 1 (CB1) and 2 (CB2), and to describe their cellular distribution in the urinary bladder of cats. Samples were collected *post-mortem* from 11 cats with no history of lower urinary tract disease and were analyzed using reverse-transcription quantitative PCR (RT-qPCR), Western blot, and immunofluorescence. Mean fluorescence intensity was analyzed in the urothelium and detrusor muscle using a paired *t*-test (*p* < 0.05). *CNR2* transcripts were detected in both compartments, whereas *CNR1* transcripts were not detectable. Western blot detected both CB1 and CB2 bands. CB1R immunoreactivity (CB1R-IR) was sparse within the urothelium, with occasional umbrella and basal cells, and was detected in endothelial cells, intramural neurons, nerve fibers, and detrusor smooth muscle cells. CB1R-IR was significantly higher in the detrusor muscle (*p* = 0.0006). CB2R-IR showed marked staining in umbrella cells and faint staining in basal cells; it was also observed in bundles of nerve fibers, intramural neurons, detrusor smooth muscle cells, and vascular structures. CB2R-IR was significantly higher in the urothelium (*p* < 0.0001). These findings provide a molecular basis for studies investigating the endocannabinoid system as a potential therapeutic target in feline lower urinary tract disorders.

## 1. Introduction

Feline lower urinary tract diseases (FLUTD) represent a common and clinically relevant group of disorders in feline medicine, encompassing conditions such as feline idiopathic cystitis (FIC), urolithiasis, urinary tract infections, and neurogenic bladder dysfunction [1]. Among these, FIC is one of the most common ones and remains a major therapeutic challenge due to its multifactorial and incompletely understood pathophysiology [2]. It involves environmental stressors, neuroendocrine dysregulation, and alterations in bladder sensory processing [3].

In cats, the urinary bladder wall is composed of mucosa (urothelium, basement membrane, and lamina propria), a muscular layer (detrusor smooth muscle), and an outer layer (serosa/adventitia) [4,5]. The urothelium is a highly specialized stratified epithelium consisting of basal, intermediate, and superficial umbrella cells [6]. Beyond its role as a passive barrier, the urothelium is recognized as an active sensory and signaling structure capable of releasing mediators such as ATP [7], nitric oxide, cytokines, and neurotrophic factors in response to mechanical and chemical stimuli [2,8,9]. These mediators modulate afferent nerve activity and contribute to the regulation of bladder function [10,11,12,13].

The endocannabinoid system (ECS), composed of cannabinoid receptors type 1 (CB1R) and type 2 (CB2R) [14], endogenous ligands (anandamide, AEA, and 2-arachidonoylglycerol, 2-AG), and metabolic enzymes (MAGL and FAAH), has emerged as an important modulator of physiological processes including nociception [15], inflammation [16,17], and smooth muscle activity [18]. Cannabinoid receptors have been localized in the non-neoplastic urinary bladder of humans [19,20] and experimental animals [20,21] in the urothelium, detrusor muscle, and neural components. Activation of cannabinoid receptors also attenuated electrically evoked contractions of isolated human detrusor strips, supporting a functional role beyond receptor localization alone [20,22]. Bakali et al. identified cannabinoid receptors, TRPV1, and the endocannabinoid-related enzymes FAAH and NAPE-PLD in human and rat bladder tissue, although receptor distribution differed between species [20].

Mukerji et al. reported increased CB1R-immunoreactive nerve fibers in patients with painful bladder syndrome and idiopathic detrusor overactivity, with associations between nerve-fiber density and pain or urgency [23]. In another human study, it was found that urothelial *CNR1* transcript levels were increased, but detrusor expression was reduced in patients with detrusor overactivity [22].

Functional studies in rats, mice, pigs, dogs, monkeys, and humans have demonstrated that activation of cannabinoid receptors can modulate bladder contractility [24,25], inhibit afferent nerve activity [9,26], and reduce inflammation in a mouse experimental model of cystitis [27]. Studies done in mice reported reduced bladder afferent sensitization, reduced inflammation, reduced urinary frequency, or improved cystitis/overactivity with CB1/CB2 agonists, endocannabinoids, or FAAH inhibition [27,28]. Cannabinoid receptor distribution was examined in rats, monkeys, and human bladder tissue, whereas in vivo cytometric experiments were performed in rats [21]. Moreover, cannabinoid agonists inhibited nerve-evoked contractions in mouse and rat bladder preparations, while WIN55,212-2 (a synthetic cannabinoid) did not produce comparable inhibition in preparations from pigs, dogs, cynomolgus monkeys, or humans under the conditions tested [24].

These findings support the hypothesis that the ECS contributes to both normal bladder physiology and pathological conditions such as overactive bladder and painful bladder syndromes. Despite growing evidence in other species, the presence and distribution of cannabinoid receptors in the feline urinary bladder remain unexplored. Given the clinical relevance of LUTD in cats and the emerging therapeutics of cannabinoids, understanding the molecular and anatomical distribution of these targets is essential.

Thus, the aim of this study was to characterize the gene (*CNR1* and *CNR2*) and protein expression, as well as the cellular distribution, of CB1R and CB2R in the urinary bladder of cats using RT-qPCR, Western blot, and immunofluorescence techniques.

## 2. Results

### 2.1. Quantitative Real-Time PCR for CNR1, CNR2

In all fixed and RNALater-preserved urothelium and detrusor samples, the reference gene YWHAZ was never detectable, even though it showed different levels of expression depending on the sampling method (Ct RNALater-preserved samples 21.3 ± 0.42; Ct paraformaldehyde 4% fixed samples 33.3 ± 2.1; mean ± SD). On the contrary, cypA was detectable only in RNALater-preserved samples, with a lower expression relative to YWHAZ and with a great variability among samples (Ct RNALater-preserved samples 30.1 ± 2.6; mean ± SD). Considering these findings, the reference gene utilized for qPCR data normalization was YWHAZ.

**Figure 1:**
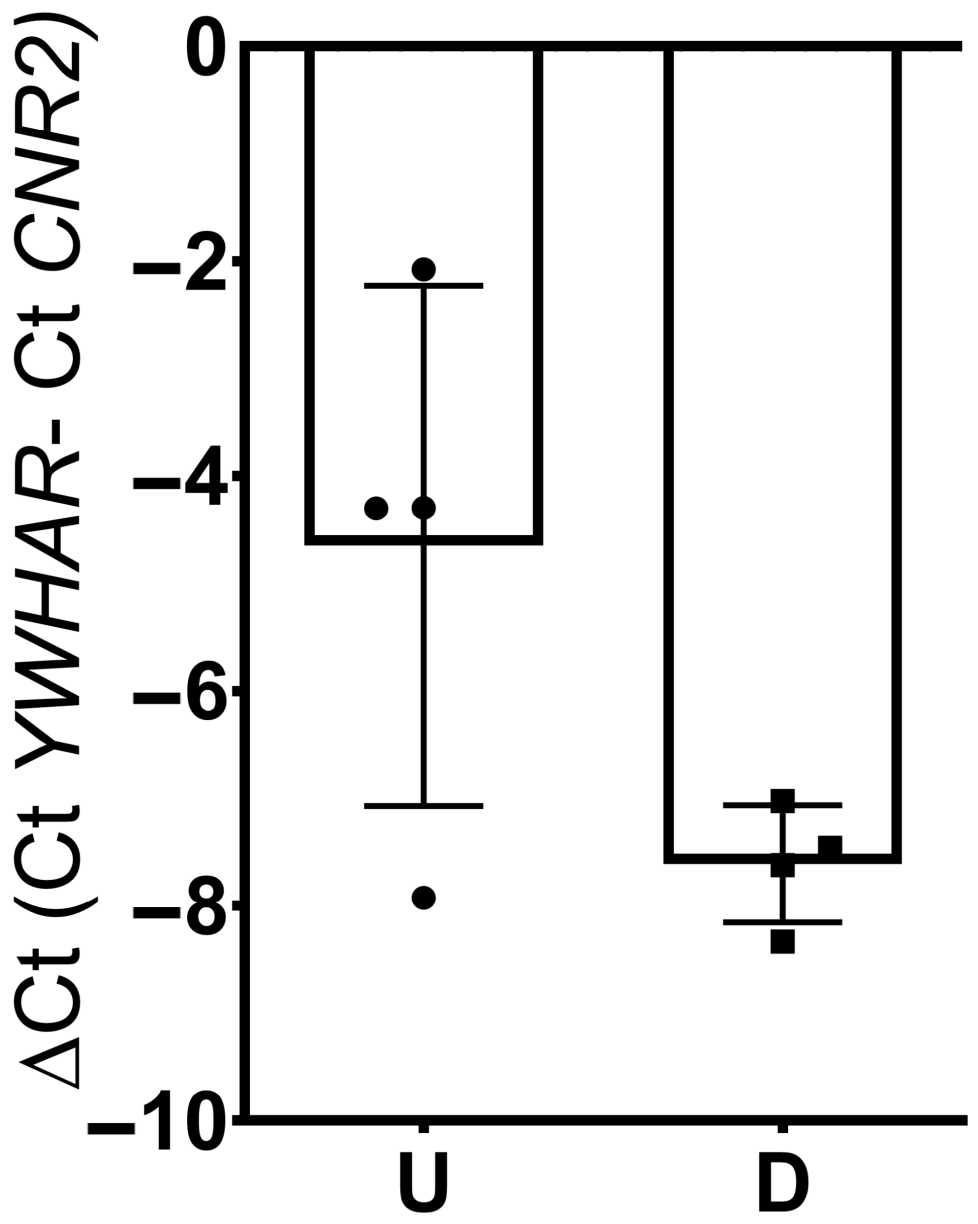
Expression of *CNR2* in feline urinary bladder (urothelium—U and detrusor—D portions) in RNALater-preserved samples. The values were reported as *ΔCt* = (*C*t reference gene − *C*t gene of interest) (mean ± standard deviation). The *ΔCt* value correlates directly with the expression level (*ΔCt* values very negative, lower expression; *ΔCt* values less negative, higher expression). Normal distributions were evaluated by means of Shapiro–Wilk tests (U *p* = 0.5289; D *p* = 0.8097), while the equality of variances in the two groups was assessed by means of Levene’s test (F value = 0.0353). No significant statistical difference (*p* < 0.05) was observed between urothelium (U) and detrusor (D) *CNR2* gene expression (Paired *t*-test, *p*-value = 0.0886). GraphPad Prism v.8 (GraphPad Software Inc., San Diego, CA, USA).

Quantitative real-time PCR of the genes of interest showed the presence of the *CNR2* transcripts in all subjects in the urothelium (U) and detrusor (D) portions of RNALater-preserved samples (*n* = 4; Figure 1), while *CNR1* was never detectable. In fixed samples, *CNR1* and *CNR2* were not detectable.

### 2.2. Western Blot

**Figure 2:**
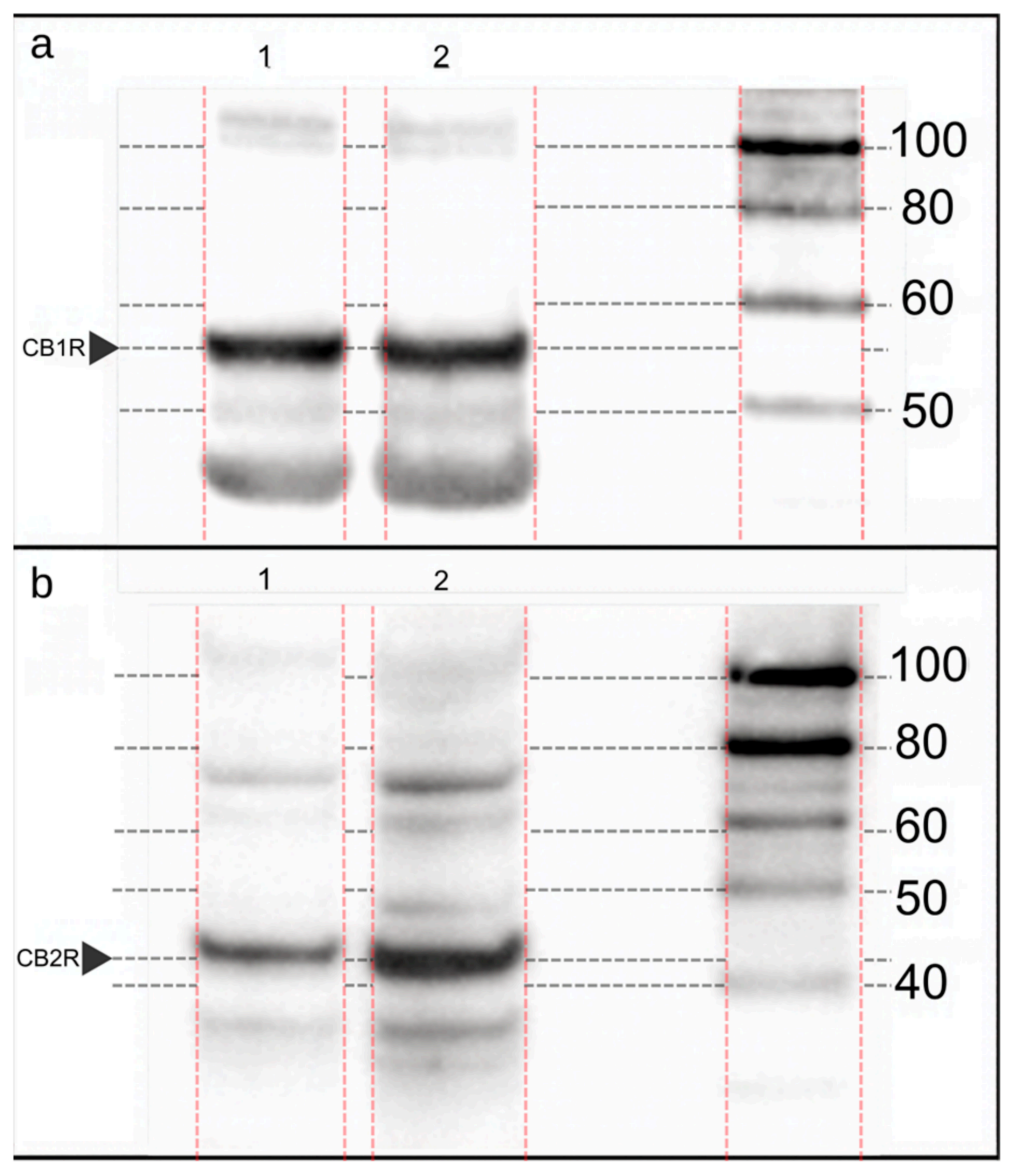
Representative image of Western blot analysis showing the antibodies’ reactivity at molecular weights compatible with the expected proteins. The rabbit anti-cannabinoid receptor 1 (Abcam, Cambridge, UK, (**a**)) and rabbit anti-cannabinoid receptor 2 (Thermo Fisher, Waltham, MA, USA, (**b**)). Each antibody showed a major band (arrowhead) close to the expected molecular weight. (**a**) Lane 1 = 20 µg cat whole bladder, Lane 2 = 40 µg cat whole bladder; Arrowhead points to the CB1R band, 50–60 kDa. (**b**) Lane 1 = 10 µg cat whole bladder, Lane 2 = 30 µg cat whole bladder; Arrowhead points to the CB2R band, 40–50 kDa. The images of the different immunoblots were slightly adjusted in brightness and contrast to match their backgrounds.

Western blot analysis of CB1R revealed a band of ~50 kDa (52,887 kDa = theoretical molecular weight of CB1R in cats). Western blot analysis of CB2R revealed a band of ~40 kDa (theoretical molecular weight of CB2R in cats is 30–56 kDa [29]) (Figure 2).

### 2.3. Immunofluorescence

#### 2.3.1. Cannabinoid Receptor Type 1 (CB1R)

**Figure 3:**
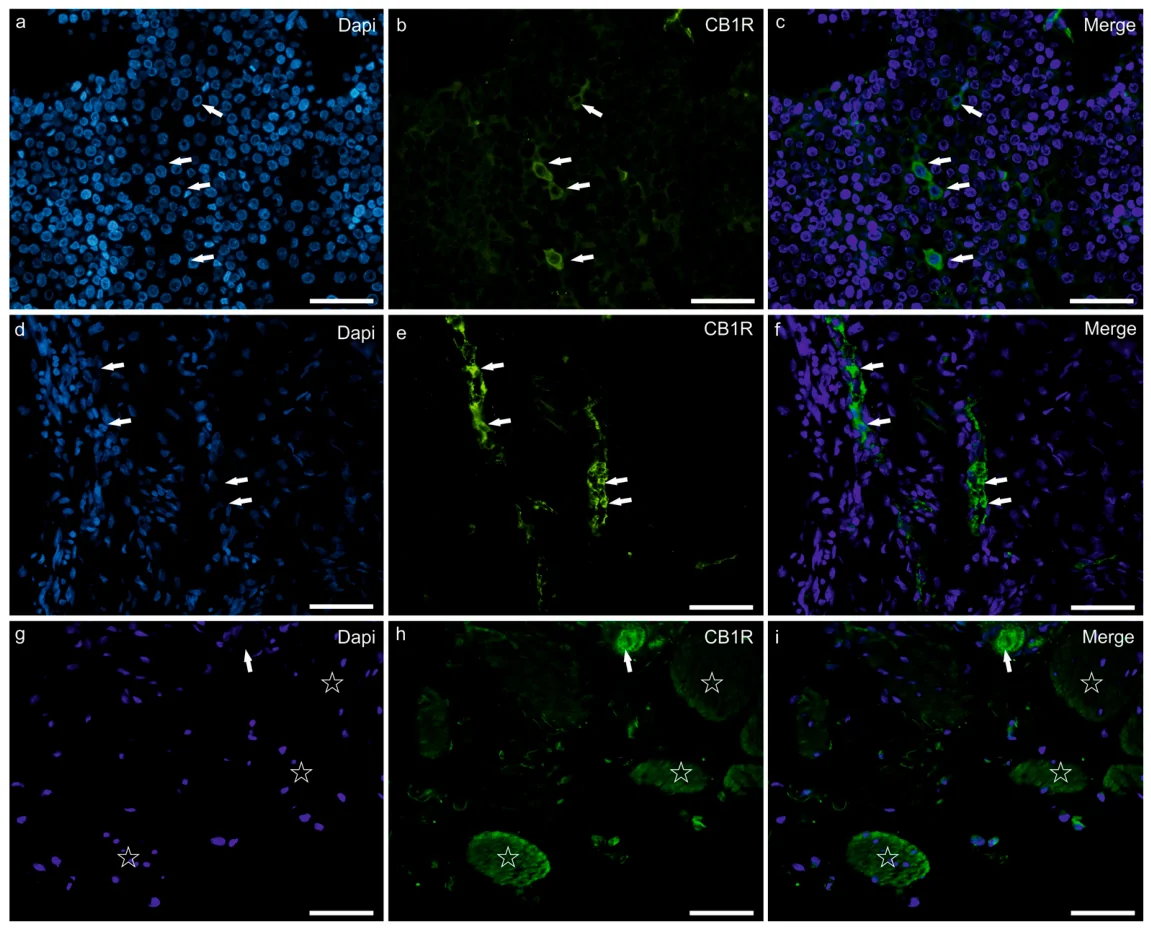
Photomicrographs of cat urinary bladder cryosections showing immunofluorescence for the cannabinoid receptor type 1 (CB1R). (**a**–**c**) The arrows indicate a few umbrella cells of the urothelium showing moderate-to-bright CB1R immunoreactivity. The cryosection is cut parallel to the epithelial surface (tangential cryosection), allowing better visualization of the few CB1R-immunoreactive cells. (**d**–**f**) The white arrows indicate some endothelial cells of two different submucosal blood vessels showing bright CB1R immunoreactivity. (**g**–**i**) The stars indicate three bundles of nerve fibers cut in cross-section showing moderate CB1R immunoreactivity;the white arrows indicate the endothelial cells of a blood vessel showing bright CB1R immunoreactivity. Scale bar = 50 µm.

In the urothelium, CB1R immunoreactivity (CB1R-IR) was largely absent. Only occasional isolated umbrella and basal cells exhibited faint to moderate cytoplasmic staining (Figure 3a–c).

Within the detrusor muscle, CB1R-IR was faintly detected in smooth muscle cells.

In the vascular compartment, moderate to bright CB1R-IR was identified in endothelial cells (Figure 3d–f) and smooth muscle cells of arterial walls.

Intramural neurons and bundles of nerve fibers displayed moderate cytoplasmic CB1R-IR (Figure 3g–i).

**Figure 4:**
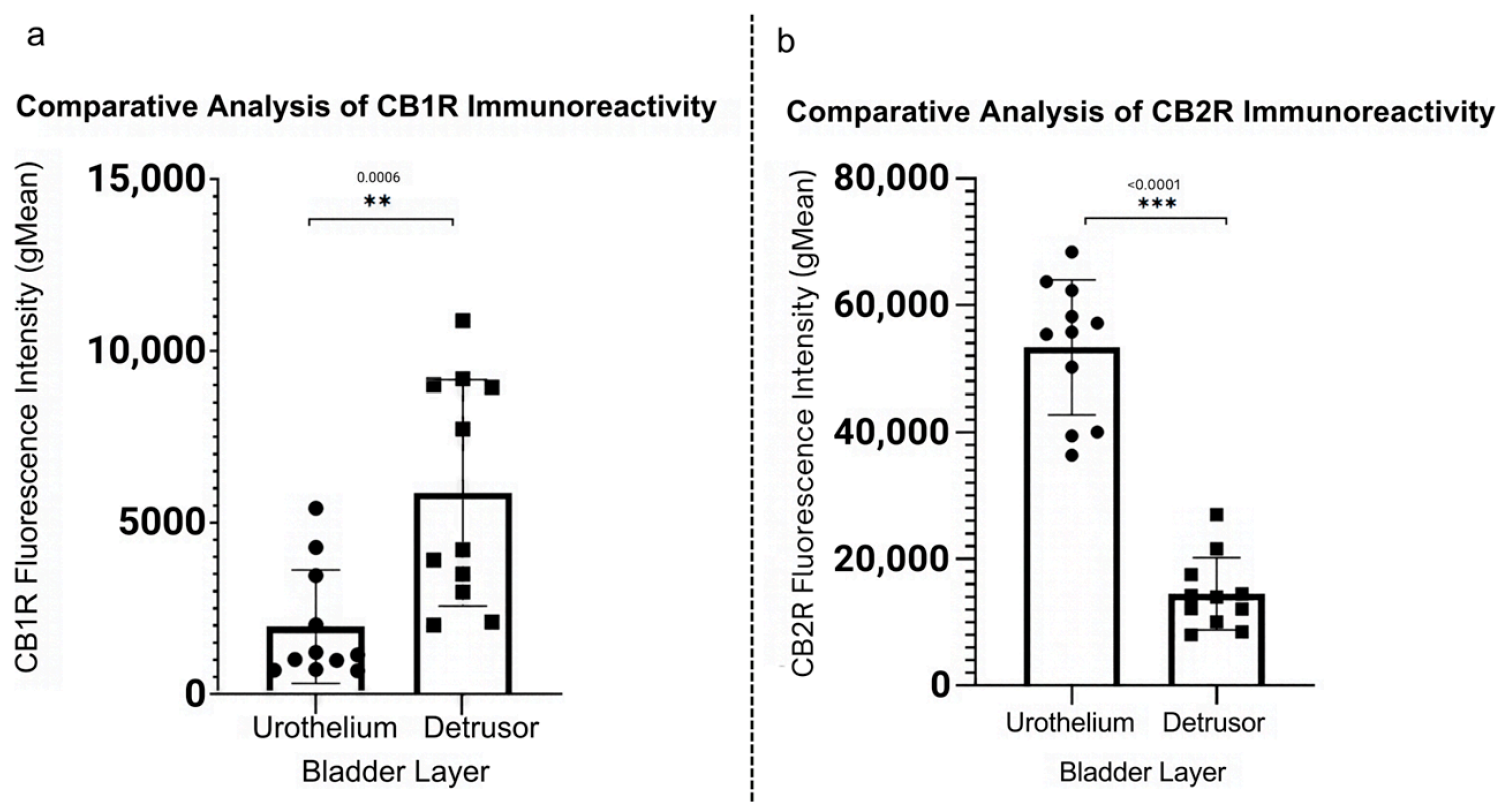
Quantitative comparison of CB1R and CB2R immunoreactivity intensity (gMEAN) in the urothelium and detrusor muscle of the urinary bladder of cats (*n* = 11). (**a**) CB1R immunoreactivity was significantly higher in the detrusor muscle than in the urothelium (paired *t*-test, *p* = 0.0006). (**b**) CB2R immunoreactivity was significantly higher in the urothelium than in the detrusor muscle (paired *t*-test, *p* < 0.0001). Bars represent mean ± SD and dots represent individual animals. “**”: *p* = 0.0006. “***”: *p* <0.0001.

The intensity of CB1R-IR was significantly higher in the detrusor muscle (mean ± SD: 5860 ± 3293 gMEAN; median: 4213) than in the urothelium (1966 ± 1656 gMEAN; median: 1143), the paired *t*-test (*t* (10) = 4.938) with *p* = 0.0006. The mean paired difference was 3894 gMEAN (95% CI: 2137–5651) (Figure 4a).

#### 2.3.2. Cannabinoid Receptor Type 2 (CB2R)

**Figure 5:**
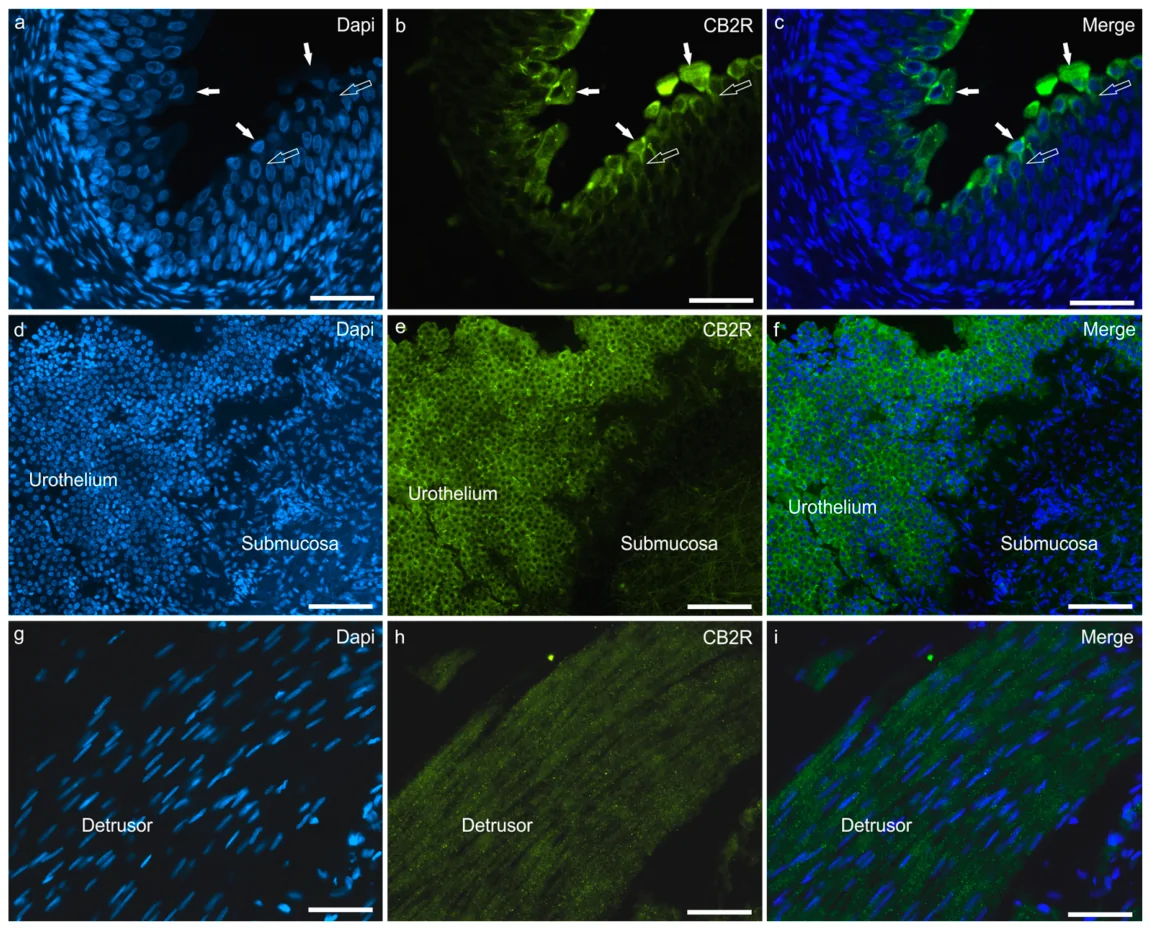
Photomicrographs of cat urinary bladder cryosections showing immunofluorescence for the cannabinoid receptor type 2 (CB2R). (**a**–**c**) The white arrows indicate the superficial large-sized umbrella cells of the urothelium showing bright CB2R immunoreactivity; the open arrows indicate their short cytoplasmic processes directed toward the intermediate cell layer. (**d**–**f**) The cryosection is cut parallel to the epithelial surface (tangential cryosection), allowing better visualization of the superficial umbrella cells, all showing bright CB2R immunoreactivity. (**g**–**i**) The smooth muscle cells of the detrusor muscle showed faint-to-moderate CB2R immunoreactivity. Scale bar = 50 µm.

In the urothelium, CB2R-IR was prominently observed in the cytoplasm of umbrella cells, displaying a distinct and consistent distribution pattern. These cells, characterized by their large size and polygonal or pear-shaped morphology, were markedly larger than cells of the intermediate and basal layers (Figure 5a–f). Cannabinoid receptor 2 immunolabeling extended into short cytoplasmic processes directed toward the intermediate cell layer, although these processes were not seen spanning beyond one to two cell layers. The intermediate cell layers exhibited faint CB2R-IR, with a gradient of expression decreasing from the superficial to the deeper layers. The most superficial intermediate cells showed a granular staining pattern, frequently localized in the perinuclear cytoplasm. Basal cells were largely negative, with only occasional cells showing faint to moderate immunoreactivity.

**Figure 6:**
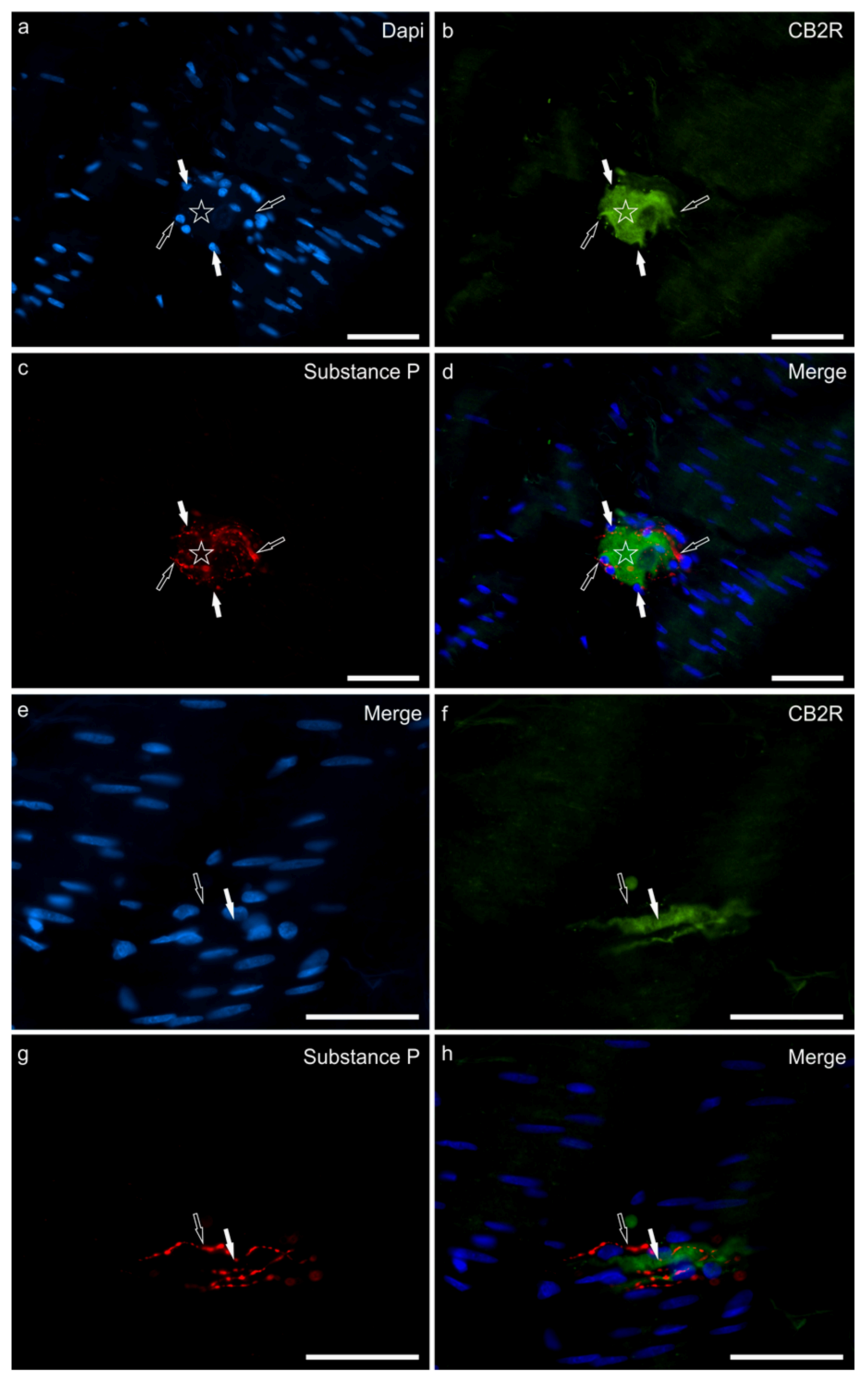
Photomicrographs of cat urinary bladder cryosections showing immunofluorescence for cannabinoid receptor type 2 (CB2R) (**b**) and the neuronal marker substance P (SP) (**c**). (**a**–**d**) The star indicates an intramural neuron of the urinary bladder showing bright CB2R immunoreactivity. The open arrows indicate varicosities along two nerve fibers showing bright SP immunoreactivity, which encircle the neuronal cell body. The white arrows indicate the nuclei of SP-negative satellite glial cells. (**e**–**h**) The white arrow indicates an elongated cell resembling an interstitial or myofibroblast-like cell, which shows moderate CB2R immunoreactivity (**f**). The open arrow indicates an SP-immunoreactive nerve fiber (**g**) in close association with the cell. Scale bar = 50 µm.

In the detrusor muscle and vascular structures, CB2R-IR ranged from faint to moderate intensity (Figure 5g–i). Bright CB2R-IR was observed in intramural neurons and nerve fibers. These neuronal elements were frequently found in close association with SP-positive varicosities (Figure 6a–d). Additionally, a small population of elongated cells within the detrusor, often located near SP-immunoreactive nerve fibers, exhibited moderate CB2R-IR (Figure 6e–h). Based on their morphology and anatomical distribution, these cells may putatively represent interstitial cells or myofibroblast-like cells; however, no specific markers were used to confirm their identity.

The intensity of CB2R-IR was significantly higher in the urothelium (mean ± SD: 53,357 ± 10,634 gMEAN; median: 55,740) than in the detrusor muscle (14,499 ± 5709 gMEAN; median: 13,984), the paired *t*-test (*t* (10) = 10.71) with a *p* < 0.0001. The mean paired difference was 38,858 gMEAN (95% CI: 30,771–46,945) (Figure 4b).

## 3. Discussion

The present study provides the first characterization of cannabinoid receptor expression in the domestic cat urinary bladder, demonstrating a differential distribution of CB1R and CB2R across urothelial, muscular, vascular, and neural compartments. These findings establish an anatomical basis for the involvement of the ECS [30] in the bladder physiology of cats.

Across species, the ECS has been identified within the urinary tract, with increasing evidence supporting its role in the regulation of bladder function. Previous studies in humans [22], rodents [20,21], and non-human primates [21] demonstrated the presence of cannabinoid receptors in the urothelium, detrusor muscle, and neural elements; their relative distribution and functional roles appear to vary among species. The present findings agree with these reports, particularly with results describing a predominance of CB2R expression within the urothelium compared to the detrusor [21,31,32].

In the present study, molecular analyses revealed distinct patterns of gene expression (mRNA) for *CNR1* and *CNR2* in the feline urinary bladder. No detectable gene expression was identified for either receptor when using paraformaldehyde-fixed samples, while in all the RNALater-preserved samples *CNR2* was measurable (*n* = 4). This finding is consistent with previous literature demonstrating that formalin fixation can lead to RNA fragmentation and chemical modification, ultimately compromising nucleic acid integrity and reducing the reliability of downstream gene expression analyses [33,34,35]. Therefore, the absence of detectable transcripts in formalin-fixed samples can be attributable to RNA degradation rather than the true absence of gene expression. However, this observation alone does not establish fixation as the sole explanation. RNA yield or purity alone would also be insufficient to demonstrate that a low-abundance target remained amplifiable. The absence of detectable *CNR1* transcripts in RNALater-preserved samples should be interpreted as a result obtained under the analytical conditions used, rather than as definitive evidence that the gene is not expressed in the feline bladder. Potential contributors include low transcript abundance, dilution of transcripts from sparsely distributed receptor-expressing cells within bulk tissue samples, differences between the tissue regions examined, and differences in transcript and protein turnover. In the present study, sampling was made up to 4 h after death, which could also influence the deterioration of genes.

For CB1R, no measurable gene expression (*CNR1*) was detected under the assay conditions in any of the samples analyzed, despite the presence of protein expression identified by immunofluorescence and Western blot. This finding may also reflect very low levels of CB1R transcription in the feline bladder, falling below the detection threshold of conventional RT-PCR techniques. Future studies employing more sensitive methodologies, such as Digital-PCR [36] or RNA sequencing [37], may be necessary to accurately quantify *CNR1* transcripts. Additionally, cell-specific approaches, including primary cell culture of CB1R-positive compartments (such as detrusor muscle or vascular-associated cells), could help clarify its transcriptional profile.

Using fresh tissue preserved in RNA stabilization solution, *CNR2* gene expression was detected in all samples. Interestingly, a tendency toward higher *CNR2* mRNA levels was observed in the detrusor muscle compared to the urothelium, although this difference did not reach statistical significance. In contrast, immunofluorescence and protein analyses demonstrated a markedly higher CB2R protein expression in the urothelium, particularly in umbrella cells.

This apparent divergence between gene and protein expression suggests a complex regulatory mechanism governing CB2R expression in the bladder. One possible explanation is that regions with higher protein expression may exhibit lower detectable mRNA levels due to increased translation efficiency and turnover of transcripts, reflecting dynamic regulation of receptor synthesis and utilization [38]. Alternatively, post-transcriptional and post-translational mechanisms, including differences in mRNA stability, protein trafficking, and receptor recycling, may contribute to this discrepancy [39]. Similar divergences between mRNA and protein levels have been described in other biological systems [40,41].

Western blotting revealed CB1R- and CB2R- bands at approximately 50 and 40 kDa, respectively. The CB1R band was consistent with the predicted molecular mass of feline CB1R (approximately 52.9 kDa) [29,42], while the CB2R band was consistent with the approximately 30–56 kDa [29]. The additional bands may be due to differences in glycosylation, that can also contribute to electrophoretic heterogeneity [43,44]. However, receptor processing or degradation and antibody cross-reactivity cannot be excluded. Thus, the observed molecular weights support, but do not establish, receptor identity, and the additional bands cannot be assigned to specific receptor isoforms.

The present WB assay works as a preliminary and exploratory experiment to verify antibody reactivity/specificity and molecular protein weights of the studied receptors in feline bladder, but it cannot show whether both receptor signals were present in each animal or assess between-animal variability. Alternative splicing is another possible explanation for CB1R/CB2R heterogeneity, as variants with modified N-terminal sequences have been characterized in humans and mice [44,45]. Using mice, sequencing of brain cDNA identified mCB1a and mCB1b, with deletions of 39 and 62 amino acids; Western blots of transfected HEK293 cells indicated markedly reduced glycosylation of mCB1b [43]. Studies in rats and mice have identified different CB2R isoforms (CB2a, CB2b, CB2c and CB2d), with individual-, species- and tissue-dependent expression [46,47]. Although, in the authors’ experience, stronger alternative bands should be seen to justify the significant number of other proteins isoforms.

In the present study, CB1R immunoreactivity exhibited a limited and heterogeneous distribution. Cannabinoid receptor 1-IR was largely absent in the urothelium, with only occasional moderate staining in isolated cells. However, CB1R was detected in intramural neurons and nerve fibers, vascular elements, and smooth muscle cells. This pattern is consistent with previous studies in rats, pigs, monkeys, and humans [26,32,48], where CB1R is predominantly associated with neural and vascular compartments and plays a role in the modulation of neurotransmitter release and vascular tone [18,48]. The presence of CB1R-IR in detrusor-associated neural structures supports its involvement in the regulation of bladder motor function [12,21,24]. Interestingly, CB1R- and CB2R-IR cells were also observed in close association with SP-immunoreactive fibers.

In contrast, CB2R showed a marked and specific expression pattern within the urothelium, with bright immunoreactivity in umbrella cells, decreasing toward intermediate layers, and minimal expression in basal cells. The urothelium is now recognized not only as a passive barrier but as an active sensory and signaling interface capable of releasing mediators such as ATP [2,7], nitric oxide, cytokines, and neurotrophic factors in response to mechanical and chemical stimuli. During bladder filling, urothelial stretch induces ATP release, which plays a key role in activating afferent nerve pathways via purinergic receptors [49,50]. Alterations in ATP signaling have been implicated in bladder hypersensitivity and lower urinary tract disorders [2,7]. There is no evidence published of CB2R’s direct role in ATP levels during bladder activity in cats.

CB2R-IR was observed in intramural neurons and nerve fibers within the detrusor. The close anatomical association between CB2R-positive structures and SP-immunoreactive nerve fibers suggests a role in sensory modulation. Interstitial cells and myofibroblast-like cells are increasingly recognized as key regulators of bladder contractility [51] and signal integration [52,53], forming a functional syncytium with urothelial and neural elements. Experimental studies in mice and human cells have demonstrated that activation of cannabinoid receptors can modulate bladder contractility and increase the micturition threshold [32], both under normal and inflammatory conditions [28]. These effects are thought to result from a combination of peripheral and central mechanisms, including inhibition of afferent nerve activity and modulation of smooth muscle excitability [12,20,24,54].

Direct evidence for cannabinoid-dependent modulation of bladder afferent activity has been obtained principally in experimental species: Walczak et al. demonstrated CB1R-dependent inhibition of mechanically evoked bladder afferent firing in mice [26,32], while activation of CB2R selectively inhibited mucosal afferents was shown in guinea pigs [55]. These findings provide a mechanistic context for feline observations, but do not demonstrate equivalent receptor function in cats.

In humans, clinical studies have investigated cannabinoids for multiple sclerosis-associated urinary symptoms (CAMS-LUTS), such as urgency, frequency, and incontinence in patients with neurogenic bladder dysfunction [25,56,57]. Although the CAMS-LUTS study reported reductions in incontinence with oral cannabis extract or THC [57], a dedicated nabiximols (Sativex^®^) trial did not meet its primary incontinence endpoint, despite improvements in several secondary measures [58]. Moreover, the FAAH inhibitor ASP3652 did not demonstrate efficacy in a randomized trial involving 287 women with interstitial cystitis/bladder pain syndrome [59]. Therefore, the presence of cannabinoid receptors in feline bladder tissue provides a rationale for functional investigation but does not establish that cannabinoid administration would improve feline lower urinary tract disease.

This study has limitations, and it is worth addressing. The sample size was limited and restricted to *post-mortem* tissues, which may not fully represent physiological variability. Given the small sample size, no subgroup analysis was performed. Functional assays were not performed, and therefore conclusions regarding receptor activity remain speculative. The incomplete medical histories of individuals may have limited the assessment of potential drug-related effects. In addition, the wide age range (1–18 years) and the different conditions affecting the patients prior to death are possible sources of variability. In addition, H&E was done in cryosections, and not in paraffin-embedded tissue, which contributed to specific processing artifacts. Immunofluorescence introduces a separate set of potential limitations, including antibody cross-reactivity, incomplete recognition of the feline epitope, fixation-dependent epitope accessibility, and tissue autofluorescence.

## 4. Materials and Methods

### 4.1. Study Design

Observational, *post mortem* analytical, cross-sectional study.

### 4.2. Animals

The study included 11 privately owned domestic cats whose cadavers were voluntarily donated for research purposes following death or euthanasia due to conditions unrelated to the urinary tract. Only animals without a history of FLUTD (Feline Lower Urinary Tract Disease) and whose cause of death was not associated with urinary tract disorders were included. Urinary bladders were collected after necropsy, and macroscopic evaluation was done by a pathologist; only organs reported with no abnormalities and pathologic signalments were included.

Urinary bladder samples from all 11 cats were collected and fixed in 4% paraformaldehyde (paraformaldehyde-fixed 4% samples) for immunofluorescence analysis and for RNA extraction. A subgroup of four cats (cats 8–11) additionally provided not fixed samples, referred to as RNALater-preserved samples: fresh tissue samples preserved in RNALater for gene expression analysis (RT-qPCR) and separate fresh tissue samples snap-frozen in liquid nitrogen for Western blot (WB) analysis.

Detailed information regarding breed, sex, age, cause of death, and sample allocation is reported in Table 1.

### 4.3. Sample Collection and Processing

Samples were taken within 4 h *post mortem.* Carcasses were stored under controlled refrigeration at +4 °C degrees (cold chamber), up to four hours post-mortem. Following a ventral midline abdominal incision, the urinary bladder was identified, isolated from surrounding tissues, and removed by transecting the proximal urethra. The organ was subsequently processed according to the analyses to be performed. Urothelium and detrusor were separated with the use of a scalpel blade n°24, anatomic pins, and anatomic thumb forceps. Following blade incision along the ventral longitudinal axis, the urothelium was detached from the detrusor by means of dissection. The procedure was done under stereomicroscopy to facilitate the view.

Fixed samples (cats 1–11) were used for immunofluorescence and gene expression (RT-qPCR): full-thickness bladder samples from all animals (*n* = 11) were fixed in 4% paraformaldehyde in 0.1 M phosphate buffer (pH 7.2) for 48 h at 4 °C. After fixation, tissues were rinsed overnight in phosphate-buffered saline (PBS). Samples intended for immunofluorescence were cryoprotected in PBS containing 30% sucrose and 0.1% sodium azide until processing. RNALater-preserved samples (*n* = 4) for RT-qPCR were retained at −80 until RNA extraction (Table 1).

Samples (*n* = 4, cats 8–11) collected from the trigonal region of the bladder were used for gene expression analysis starting from. The urothelium and detrusor muscle were carefully dissected and immediately immersed in RNAlater solution (Thermo Fisher Scientific, Waltham, MA, USA). Samples were subsequently stored at −80 °C until RNA extraction (Table 1).

For Western blot analysis, whole bladder samples from the same four animals (*n* = 4, cats 8–11) were snap-frozen in liquid nitrogen immediately after collection and stored at −80 °C until protein extraction (Table 1).

In addition, hematoxylin and eosin (H&E)-stained cryosections of urinary bladder tissue were examined to identify histopathological abnormalities indicative of subclinical/non-diagnosed urinary bladder disease. The evaluation included urothelial integrity and morphology, epithelial hyperplasia or atypia, inflammatory cell infiltration, edema, vascular congestion, hemorrhage, fibrosis, and structural alterations of the detrusor muscle [60,61]. Bladders exhibiting inflammatory, degenerative, proliferative, or neoplastic lesions, or autolytic artifacts compromising histological interpretation, were excluded from subsequent analyses (Supplementary Figure S1). The examined photomicrographs showed preserved bladder wall architecture, with largely continuous urothelial coverage and organized detrusor smooth muscle bundles. No overt ulcerative, necrotizing, inflammatory, suppurative, or mass-forming lesions were seen.

### 4.4. RNA Extraction and Analysis of Expression of CNR1 and CNR2

For gene expression analysis, total RNA extraction was carried out from urothelium and detrusor RNALater-preserved samples (*n* = 4) and fixed samples (*n* = 11) (Table 1) using different kits.

The RNA extraction from fixed samples (4% paraformaldehyde) was performed following the manufacturer’s instructions for the kit (RNeasy FFPE kit, Code: 73504, Qiagen, Hilden, Germany) with some modifications with a few modifications, as previously [40]. The fixed samples from the urothelium and the detrusor were detached, taken, and weighed. Fifty (50) μg of urothelium (*n =* 11) and 50 μg of detrusor (*n =* 11) were collected. The samples were macerated using a bistoury blade n°22 and added to 240 μL of Buffer PKD [Proteinase K Digestion Buffer, Qiagen, Hilden, Germany], mixed well by vortexing, centrifuged for 1 min at 11,000× *g*, followed by the addition of Proteinase k (10 μL) and mixed gently. The samples were incubated at 56 °C for 3 h; every 30 min, the samples were mixed/homogenized using a micro-pestle (Eppendorf, Hamburg, Germany). The samples were then incubated at 80 °C for 15 min, and the manufacturer’s protocol was followed up to elution (20 μL).

Total RNA extraction from RNALater-preserved (*n* = 4, not fixed) samples was performed using TRI Reagent (Molecular Research Center, Inc., Cincinnati, OH, USA) and NucleoSpin RNA II (Macherey-Nagel GmbH & Co. KG, Düren, Germany) kit according to the manufacturer’s instructions. Briefly, tissue was homogenized in TRI Reagent (50 mg/ml) with an Ultra-Turrax, then 200 μL of chloroform was added to the suspension and mixed well. After incubation at room temperature (10 min), samples were centrifuged (12,000× *g* for 10 min), and the aqueous phase was recovered. An equal volume of absolute ethanol (99%) was added, and the resulting solution was applied to the NucleoSpin RNA Column (Qiagen, Hilden, Germany) as previously described [62].

After spectrophotometric quantification, the total RNA (500 ng) was reverse transcribed to complementary DNA (cDNA) using 5× iScript RT Supermix (Bio-Rad Laboratories, Inc., Hercules, CA, USA) at a final volume of 20 μL. To evaluate the gene expression profiles, RT-PCR was carried out in a CFX96 thermal cycler (Bio-Rad Laboratories, Inc.) using SYBR green detection to target the genes. Specific primers for cats were used to evaluate the gene expression for cannabinoid receptors 1 and 2 (*CNR1* and *CNR2*) and reference genes for tyrosine 3-monooxygenase/tryptophan 5-monooxygenase activation protein zeta and cyclophilin A (*YWHAZ* and *cypA/CYC,* respectively) (Table 2).

All the amplification reactions were carried out in 20 μL aliquots and analyzed in duplicate; the reaction contained 10 μL of iTaq Universal SYBR Green Supermix (Bio-Rad Laboratories Inc.), 0.8 μL of the forward and reverse primers (5 mM each) of each target gene, 2 μL cDNA, and 7.2 μL of water. The real-time procedure included an initial denaturation period of 3 min at 95 °C, 40 cycles at 95 °C for 15 s and 60 °C for 30 s, followed by a melting step with ramping from 55 °C to 95 °C at a rate of 0.5 °C/10 s. In addition to urothelium and the detrusor samples, RT-qPCR assays were also performed on transcripts from the cat’s brain as a positive control for *CNR1* and *CNR2*. Real-time efficiency was evaluated by amplification of a standardized amount of cDNA, derived from the cat’s brain (RNALater-preserved), starting from 75 ng with subsequent 5-fold dilutions (75, 15, 3, 0.6, and 0.12 ng). The efficiency showed values of 97.5% and 92.3% for *CNR1* (a) and *CNR2* (b), respectively (Supplementary Figure S2). The specificity of the amplified PCR products was confirmed by agarose gel electrophoresis and melting curve analysis. The relative expression of the genes of interest was normalized based on the reference gene. The relative mRNA expression of the gene tested was evaluated using the *ΔCt* method with *ΔCt* = (*C*t reference gene − *C*t gene of interest), which correlated directly with the expression level (*ΔCt* values very negative, lower expression; *ΔCt* values less negative, higher expression).

### 4.5. Western Blot

Western blot was performed as previously described [64]. Briefly, tissue samples (whole bladder) were collected, frozen in liquid nitrogen, and stored at −80 °C until sample processing. A pool of the four animals was made, using 12.5 mg of tissue each, with a total of 50 mg of tissue, which was then homogenized in 500 µL of SDS buffer (Tris–HCl, 62.5 mM; pH 6.8; SDS, 2%; and glycerol, 20%, Sigma-Aldrich Co., St. Louis, MO, USA) supplemented with a protease inhibitor cocktail (Sigma-Aldrich Co., St. Louis, MO, USA). Total protein content was determined by Peterson’s Modification of the Lowry Method using a Protein Assay Kit. Aliquots containing 10 to 40 µg of total protein were separated on NuPAge 4–12% bis-Tris Plus (Life Technologies Ltd., Paisley, UK) for 30 min at 200 V. The proteins were then electrophoretically transferred onto a nitrocellulose membrane by a semi-dry system (Trans Turbo Blot Bio-Rad, Hercules, CA, USA). Non-specific binding on nitrocellulose membranes was blocked with 5% milk powder in PBS-T20 (Phosphate-Buffered Saline-0.1% Tween-20) for 1 h at RT. After blocking treatment, the membranes were incubated overnight at 4 C with the primary antibodies (Table 3) (CB1R 1:100, CB2R 1:250) diluted in PBS-T20 (0.1% T-20). After washes, the blots were incubated with a goat anti-rabbit biotin-conjugate antibody (1:50,000 dilution in PBS-T20, 1 h at RT) and then with a 1:1000 dilution of an anti-biotin horseradish peroxidase (HRP)-linked antibody (45 min at RT). Immunoreactive bands were visualized using chemiluminescent substrate (Clarity Western ECL Substrate Bio Rad, Hercules, CA, USA), according to the manufacturer’s instructions. The intensity of the luminescent signal was acquired by Chemidoc Instrument (Bio-Rad, Hercules, CA, USA), and the apparent molecular weight of the resultant bands was analyzed by Quantity One Software (Version 4.6.8, Bio-Rad).

### 4.6. Immunofluorescence on Cryosections

Small pieces (1 cm × 0.5 cm) of the full-thickness wall of the urinary bladders kept in PBS-30% sucrose were transferred to a mixture of PBS-30% sucrose–azide and Optimal Cutting Temperature (OCT) compound (Histo-Line Laboratories, Pantigliate, MI, Italy) at a ratio of 1:1 for an additional 24 h before being embedded in 100% OCT in Cryomold^®^ (Sakura Finetek Europe, Alphen aan den Rijn, The Netherlands, Europe). The sections were prepared by freezing the tissues in isopentane cooled in liquid nitrogen. The cryosections (14 μm thick) of the bladder were cut on a cryostat and mounted at room temperature (RT, 22–25 °C) on polylysinated slides.

The protocol was performed as previously described [65]. The cryosections were hydrated in PBS and processed for immunostaining [62,66]. To block non-specific binding, the sections were incubated in a solution containing 20% normal donkey serum (Colorado Serum Co., Denver, CO, USA), 0.5% Triton X-100 (Sigma-Aldrich, Milan, Italy, Europe), and bovine serum albumin (1%) in PBS for 1 h at RT. The cryosections were incubated in a humid chamber overnight at RT with the antibodies directed against CB1R and CB2R (single immunostaining) or with a combination of primary antibodies (double immunostaining) (Table 3) diluted in 1.8% NaCl in 0.01 M PBS containing 0.1% sodium azide combined with 20% normal donkey serum (Colorado Serum Co., Denver, CO, USA), 0.5% Triton X-100 (Sigma-Aldrich, Milan, Italy, Europe), and bovine serum albumin (1%). After washing in PBS (3 × 10 min), the sections were incubated for 1 h at RT in a humid chamber with the secondary antibodies (Table 4) diluted in PBS and with the same previous compounds. The cryosections were then washed in PBS (3 × 10 min) and mounted in buffered glycerol at pH 8.6 with 4′,6-diamidino-2-phenylindole—DAPI (Santa Cruz Biotechnology, Santa Cruz, CA, USA). All the reactions were done in the same batch for each protein target and co-localization.

#### 4.6.1. Specificity of the Primary Antibodies

The specificities of the anti-CB1R and anti-CB2R antibodies were tested in the present study by Wb analysis.

The anti-CB1R antibody has been used in cats [67,68]. This antibody identifies the human Cannabinoid Receptor type 1 (*CNR1*), raised against the peptide within human CB1 residues 450–C-terminus. The homology between this gene and the feline gene (*Felis catus*) is 98.1% (https://www.uniprot.org/blast) (accessed on 15 October 2026).

The anti-CB2R antibody identifies the human Cannabinoid Receptor type 2 (*CNR2*), raised against the first 33 amino acids of human CB2. The homology between this gene and the feline gene (*Felis catus*) is 83.6% (https://www.uniprot.org/blast) (accessed on 15 October 2026).

#### 4.6.2. Specificity of the Secondary Antibodies

The specificity of the secondary antibodies was tested by applying them to the sections after the omission of the primary antibodies. No stained cells were detected after omitting the primary antibodies.

#### 4.6.3. Quantitative and Qualitative Microscopic Analysis of CB1R and CB2R Immunofluorescence

Quantitative analysis of immunoreactivity of CB1R and CB2R in the urothelium and the detrusor was conducted. For each animal, five random photomicrographs of the urothelium and of the detrusor muscle were acquired at high magnification (400×) using the same exposure time for all the images and analyzed blindly for positive immunoreactive staining. From each animal, a median value of signal intensity (gMEAN) for each target was assessed. gMEAN is the median value of immunofluorescence intensity of the green fluoroscope. ImageJ software (ImageJ, version 1.52t, National Institutes of Health, Bethesda, MD, USA) was used to analyze the signal intensity of each image using the Color Histogram (gMEAN) tool of the software. All images were background corrected using NIS Elements software BR 4.20.01 (Nikon Instruments Europe BV, Amsterdam, The Netherlands, Europe).

The qualitative immunofluorescence of the antibodies and their cellular distribution were evaluated. The intensity of the expression was evaluated as faint, moderate, or bright on microscopy observation. The preparations were examined using a Nikon Eclipse Ni microscope equipped with the appropriate filter cubes. The images were recorded with a DS-Qi1Nc digital camera and NIS Elements software BR 4.20.01 (Nikon Instruments Europe BV, Amsterdam, The Netherlands, Europe).

### 4.7. Statistical Methods

For each cat, the mean fluorescence intensity values from the urothelium and detrusor muscle were compared as paired observations (*n* = 11 pairs). Normality of the paired differences between urothelium and detrusor muscle was assessed using the Shapiro–Wilk test. No significant deviation from normality was detected for either CB1R (W = 0.889, *p* = 0.136) or CB2R (W = 0.897, *p* = 0.168). Thus, differences in immunofluorescence intensity between the two regions were analyzed using paired *t*-tests. For gene (RT-qPCR) analysis, a paired *t*-test was used (*n* = 4 pairs). Statistical significance was set at *p* < 0.05. Statistics were performed using GraphPad Software Version 8.0.

Generative AI was exclusively used to build the graphical abstract, under the supervision of the authors, and the results were curated by all the authors and approved. No generative artificial intelligence (GenAI) has been used to generate text, data, or statistical graphics, or to assist in study design, data collection, analysis, interpretation, or to generate scientific data.

## 5. Conclusions

The present study demonstrates, for the first time, the presence and differential distribution of cannabinoid receptors CB1R and CB2R in the feline urinary bladder, as well as the gene *CN2R* expression, but not *CNR1*. The use of fixed samples to perform RT-qPCR is not advisable due to potential degradation of genes. The marked expression of CB2R in the urothelium and neural elements points to a role in epithelial signaling and sensory modulation, while CB1R is associated with neural and vascular regulation. These findings point to the involvement of the ECS in cat bladder physiology. In addition, standardized and species-specific molecular methodologies for cats are needed.

## Figures and Tables

**Table 1:** Signalment, cause of death, and sample allocation of the 11 cats included in the study. Animals ranged from 1 to 18 years of age (mean ± SD: 8.5 ± 5.6 years; median: 9 years).

Nº	Breed	Sex	Age (Years)	Reason of Death	Fixed IF	Fixed RT-qPCR	RNA Later RT-qPCR	Fresh (Snap Frozen) WB
1	European Shorthair	F	5	Road traffic accident	Yes	Yes	No	No
2	European Shorthair	F	9	Acute Heart Failure	Yes	Yes	No	No
3	European Shorthair	F	16	Osteosarcoma	Yes	Yes	No	No
4	European Shorthair	M	1	Road traffic accident	Yes	Yes	No	No
5	European Shorthair	M	3	Diabetes Mellitus	Yes	Yes	No	No
6	Maine Coon	M	11	Pneumonia	Yes	Yes	No	No
7	Ragdoll	F	3	Ketoacidosis + Septic Shock	Yes	Yes	No	No
8	European Shorthair	F	18	Multiple skin tumors	Yes	Yes	Yes	Yes
9	European Shorthair	F	13	Intestinal neoplasia	Yes	Yes	Yes	Yes
10	Persian	M	10	Pancreatitis	Yes	Yes	Yes	Yes
11	European Shorthair	M	4	Congenital hypertrophic cardiomyopathy	Yes	Yes	Yes	Yes

Abbreviations: F—female; M—male; IF—Immunofluorescence; RT qPCR—Reverse Transcription Quantitative Polymerase Chain Reaction; WB—Western blot.

**Table 2:** Primer sequence, predicted product size, and accession number (AN) used for quantitative real-time PCR analysis.

Gene	Sequence (5′–3′)	PCR (bp)	AN	Reference
*CNR1*	For: AATTTCCTTTAACTTCCTTTAG Rev: TCCTCATTCTCCTTGTAG	142	U94342	Present study
*CNR2*	For: TCAGCATTTGTCTCCTAC Rev: AGCCCAGCAGATAGTTAT	100	XM_045035285	Present study
*YWHAZ*	For: GAAGAGTCCTACAAAGACAGCACGC Rev: AATTTTCCCCTCCTTCTCCTGC	116	EF458621	[63]
*CYC*	For: CCTTCTGTAGCTCGGGTCAG Rev: CTTGGAGGGGAGGTAAGGAG	113	AY029366	[63]

**Table 3:** Primary antibodies used in the study. Dilution is for immunofluorescence experiments in cryosections.

Primary Antibody	Host	Code	Dilution	Source
CB1R	Rabbit	Ab23703	1:100	Abcam
CB2R	Rabbit	PA1-744	1:250	Thermo Fisher
Substance P	Rat	10-515A	1:500	Fitzgerald

Primary antibody Suppliers: Abcam, Cambridge, UK; Dako Cytomation, Golstrup, Denmark; Fitzgerald Industries Int., Inc., Concord, MA, USA; Santa Cruz Biotechnology, California, USA; Thermo Fisher Scientific, Waltham, MA, USA; Monosan, Noord-Brabant, The Netherlands. Abbreviations: CB1R—Cannabinoid Receptor Type 1; CB2R—Cannabinoid Receptor Type 2.

**Table 4:** Secondary antibodies used in the study. Dilution is for immunofluorescence experiments in cryosections.

Secondary Antibody	Host	Code	Dilution	Source
Anti-rabbit IgG Alexa-488	Donkey	A-21206	1:1000	Thermo Fisher
Anti-rat IgG Alexa-594	Donkey	A-21209	1:500	Thermo Fisher

Secondary antibody Suppliers: Thermo Fisher Scientific, Waltham, MA, USA.

## Data Availability

The datasets presented in this study can be found in online repositories. The names of the repository/repositories and accession number(s) can be found in the article/Supplementary Materials. The datasets for this study can be found in AMSACTA from the University of Bologna (https://amsacta.unibo.it/id/eprint/8873) (accessed on 22 July 2026)

## Supplementary Materials

The following supporting information can be downloaded at: https://www.mdpi.com/article/10.3390/ijms27219910/s1.

## Abbreviations

The following abbreviations are used in this manuscript:

ECS	Endocannabinoid System
CB1R	Cannabinoid Receptor type 1
CB2R	Cannabinoid Receptor type 2
WB	Western Blot
LUTD	Lower Urinary Tract Disease
SP	Substance P
PBS	Phosphate-buffered saline
OCT	Optimal Cutting Temperature compound
FIC	Feline Idiopathic Cystitis
MAGL	Monoacylglycerol Lipase
FAAH	Fatty Acid Amide Hydrolase
2-AG	2-Arachidonoylglycerol
ATP	Adenosine Triphosphate
RT-qPCR	Reverse transcription quantitative polymerase chain reaction
IF	Immunofluorescence
IR	Immunoreactivity
RT	Room Temperature
DAPI	4′,6-diamidino-2-phenylindole
RNA	Ribonucleic Acid
mRNA	Messenger Ribonucleic Acid
FLUTD	Feline Lower Urinary Tract Disease
